# Cementless curved endoprosthesis stem for distal femoral reconstruction in a Chinese population: a combined anatomical & biomechanical study

**DOI:** 10.1186/s12891-022-05801-z

**Published:** 2022-09-08

**Authors:** Xin Hu, Minxun Lu, Yitian Wang, Yang Wen, Linyun Tan, Guifeng Du, Yong Zhou, Yi Luo, Li Min, Chongqi Tu

**Affiliations:** 1 Department of Orthopedics, Orthopedic Research Institute, West China Hospital, Sichuan University. No, 37 Guoxuexiang, Chengdu, 610041 Sichuan People’s Republic of China; 2 Department of Model Worker and Innovative Craftsman, West China Hospital, Sichuan University, No. 37 Guoxuexiang, Chengdu, Sichuan 610041 People’s Republic of China

**Keywords:** Uncemented, Press–fit, Stem, Short, Distal femur, Finite element analysis

## Abstract

**Background:**

The endoprosthetic knee reconstruction using a current universal femoral stem might not be suitable for local population due to the anatomical difference between Chinese and Western populations. We measured the anatomical parameters of Chinese femurs as reference for stem design, and proposed a cementless, curved, short endoprosthesis stem for the reconstruction of distal femur. This study analyzed the biomechanical performance of the newly designed stem aimed at the identification of better operative strategy.

**Methods:**

The CT–scanning data of femurs derived from 96 healthy Chinese volunteers were imported into the Mimics software, and a segmental measurement strategy was applied to evaluate the radius of curvature (ROC) of the femoral medullary cavity. Then, 4 kinds of endoprosthetic replacement models were created based on the measurement results. Model A: the distal tumor resected femora + straight stem A; Model B: the distal tumor resected femora + curved stem B; Model C: the distal tumor resected femora + curved stem C; Model D: the distal tumor resected femora + curved stem D. Finally, the mechanical difference among these models were compared by finite element analysis.

**Results:**

The mean femoral ROC of Segment_1, 2, 3, 4, 5_ measured in the present study was 724.5 mm, 747.5 mm, 1016.5 mm, 1286.5 mm, and 1128 mm, respectively. Based on the femoral ROC of Segment_2,_ the stem ROC of the curved stem B, C, and D was designed as 475 mm, 700 mm, and 1300 mm, respectively. Generally, all endoprosthetic replacement models showed a normal–like stress distribution on the femurs. However, compared to the straight stem, the biomimetic curved stem showed better biomechanical performance both in terms of reducing the extent of the stress shielding of the femur and in terms of minimizing the stress distribution of the implant.

**Conclusions:**

The uncemented, curved, short stem with suitable ROC can perfectly match the Chinese femoral canal morphology which has better mechanical properties than the conventional femoral stem. Thus, this newly designed femoral stem might be an optimized method for treatment of malignant femoral tumours in the Chinese populations in the case that the numerical results are supported by future experimental studies.

## Introduction

Tumour endoprostheses has evolved over the last decades, and endoprosthetic reconstruction combined with chemotherapy has made limb–salvage surgery possible, which significantly decreases the rate of amputations and increases lifespan of patients with malignant bone sarcoma [1, 2]. However, the endoprosthetic reconstruction is frequently associated with many complications, including aseptic loosening, periprosthetic infection, and mechanical failure [3]. Among these complications, aseptic loosening of femoral stem is one of the main causes of knee revision surgery [4]. As a complex and multifactorial event, the aseptic loosening is the result of a combination of various factors, including particles wear, stress shielding, micromotion, and so on [5]. However, it is believed that the loosening of the femoral stem tends to be a mechanical event [6]. Thus, reasonable fixation method and good mechanical stability are of great importance for reducing the aseptic loosening rate [7].

Currently, there are two methods commonly used: cemented fixation and uncemented fixation. The cemented stem can provide immediate stability which allows early weight bearing postoperatively [8], but its unacceptable rate of aseptic loosening limited its application. In contrast, the uncemented press–fit fixation has been associated with a lower loosening rate due to its biological fixation [9–11]. In realizing the success of uncemented fixation, excellent primary stability is an essential prerequisite. Thus, the uncemented stem demands an optimal geometric fit between the stem and the femoral canal to ensure tight contact and primary stability [12]. Because of the specific anatomical features of the femoral canal which has an antecurvation in the sagittal plane, the curved stem should theoretically be able to better match the medullary cavity compared to the straight stem [11]. However, most of commercially available prosthetic devices provided a straight femoral stem design. Even though a few knee reconstruction systems, including Modular Universal Tumor and Revision System (MUTARS, Implantcast GmbH, Buxtehude, Germany), the Segmental System (Zimmer Inc., Warsaw, IN, USA) provided curved stem [9, 13], these prostheses are designed and manufactured in Europe and North American which are tailored to their anatomical features [14, 15]. Therefore, a curved press–fit stem suitable for Chinese is urgently needed.

To date, fewer studies have investigated the anatomical antecurvation of Chinese femora, and no corresponding curved stem has been developed for Chinese. This study aims to investigate the Chinese femoral canal and to design a biomimetic curved stem. In addition, the mechanism and biomechanical properties of this alternative prosthesis were analyzed and discussed by finite element analysis (FEA).

## Methods

### Anatomical study

Adult Chinese volunteers with no evidence of lower extremity trauma, congenital deformity of knee, hip, and femur, deformity from prior trauma or intervention, and femoral or pelvic implants were included. Based on these criteria, a total of 96 healthy Chinese volunteers were included at our institution between December 2016 and March 2021. All anticipants underwent femur three–dimensional computed tomography (3D–CT) scans, and the imaging and demographic data (age, gender, height, and weight) were collected. This study was authorized by our Ethical Committee, and the written informed consent was obtained from all volunteers.

#### Coordinate system calibration and data collection protocol

The femurs were measured by 3D–CT, all CT scans were performed by Philips Brilliance 64 CT system (Philips Healthcare, Netherlands). Neutral position of lower limbs was suggested. The coverage of CT scan was from the anterior superior iliac spine inferior margin to the middle of the tibiofibular joint. Both slice thickness and increment were set as 0.4 mm to ensure the accuracy. The rest parameters of scanning were as follows: kilovolt peak, 120 kV; X–Ray tube current, 240 mA.

**Fig. 1:**
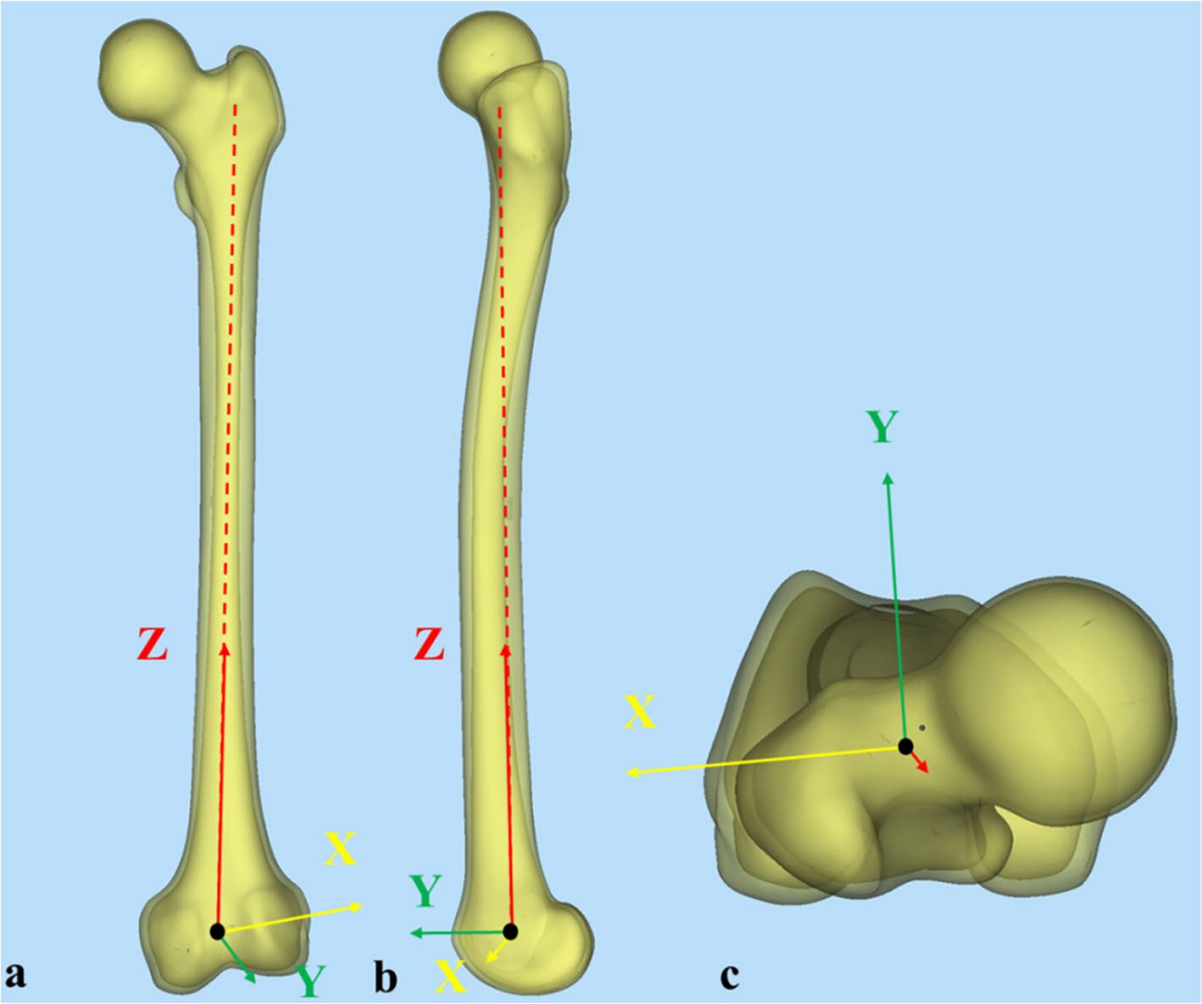
Diagram of the femur–based (FB) coordinate system: **a** Front view. **b** Side view. **c** Top view

Next, all CT data were further imported into Mimics Research 20.0 (Materialise Corp., Belgium), and a femur–based (FB) coordinate system was also established to precisely measure the radius of curvature (ROC) of collected femurs [16, 17]. In the FB coordinate system, the hip–joint center of rotation (HJC) was defined as the origin O. The Z–axis was strictly parallel to the longitudinal axial of femoral shaft, pointing proximally. Meanwhile, the X–axis was parallel to the surgical transepicondylar axis (sTEA), pointing laterally. Finally, the Y–axis was perpendicular to both X–axis and Z–axis, pointing anteriorly. In addition, the X–Z, X–Y, and Y–Z planes were respectively defined as the coronal, horizontal, and sagittal planes in the FB coordinate system. (Fig. 1a-c).

#### Measurement of the radius of curvature

**Fig. 2:**
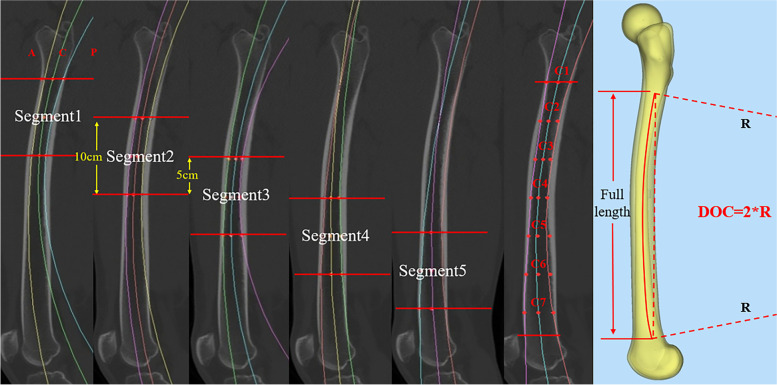
The segmental measurement strategy in sagittal plane

A segmental measurement strategy was applied to evaluate the ROC variation in different segments of the femoral medullary cavity (Fig. 2), and the coverage of each segment was defined in Table 1.

**Table 1:** The coverage of each of segments in sagittal plane

Segment	Length (cm)	Coverage (start/end)
Segment_1_	10	the lesser trochanter/the 10 cm below the lesser trochanter
Segment_2_ Segment_3_ Segment_4_ Segment_5_	10 10 10 10	the 5 cm below the lesser trochanter/the 15 cm below the lesser trochanter the 10 cm below the lesser trochanter/the 20 cm below the lesser trochanter the 15 cm below the lesser trochanter/the 25 cm below the lesser trochanter the 20 cm below the lesser trochanter/the 30 cm below the lesser trochanter

On each segment, the center of the medullary canal was identified and named as C_1_, C_2_, C_3_, C_4_, C_5_, C_6_, C_7_ from proximal to distal. An imaginary curved line is drawn through the adjacent three center points, which must be parallel to central axis of the corresponding femoral segment. The radius measurement tool of Mimics software was applied to measure the ROC of each segment, named ROC_123_, ROC_234_, ROC_345_, ROC_456_, ROC_567_. For instance, ROC_123_ was determined by the position of C_1_, C_2_, and C_3_, and represented the curvature radius of Segment_1_. (Fig. 2).

#### Statistical analysis

Data analyses were performed using IBM SPSS Statistics 20.0 (IBM SPSS, Armonk, NY). The normality of the continuous data was checked by the one sample Kolmogorov–Smirnov test. The ROC data of each segment were compared using One–way analysis of variance (ANOVA). A *p* < 0.05 was considered statistically significant.

### Biomechanical study

#### The normal femur and the osteotomy simulation

**Fig. 3:**
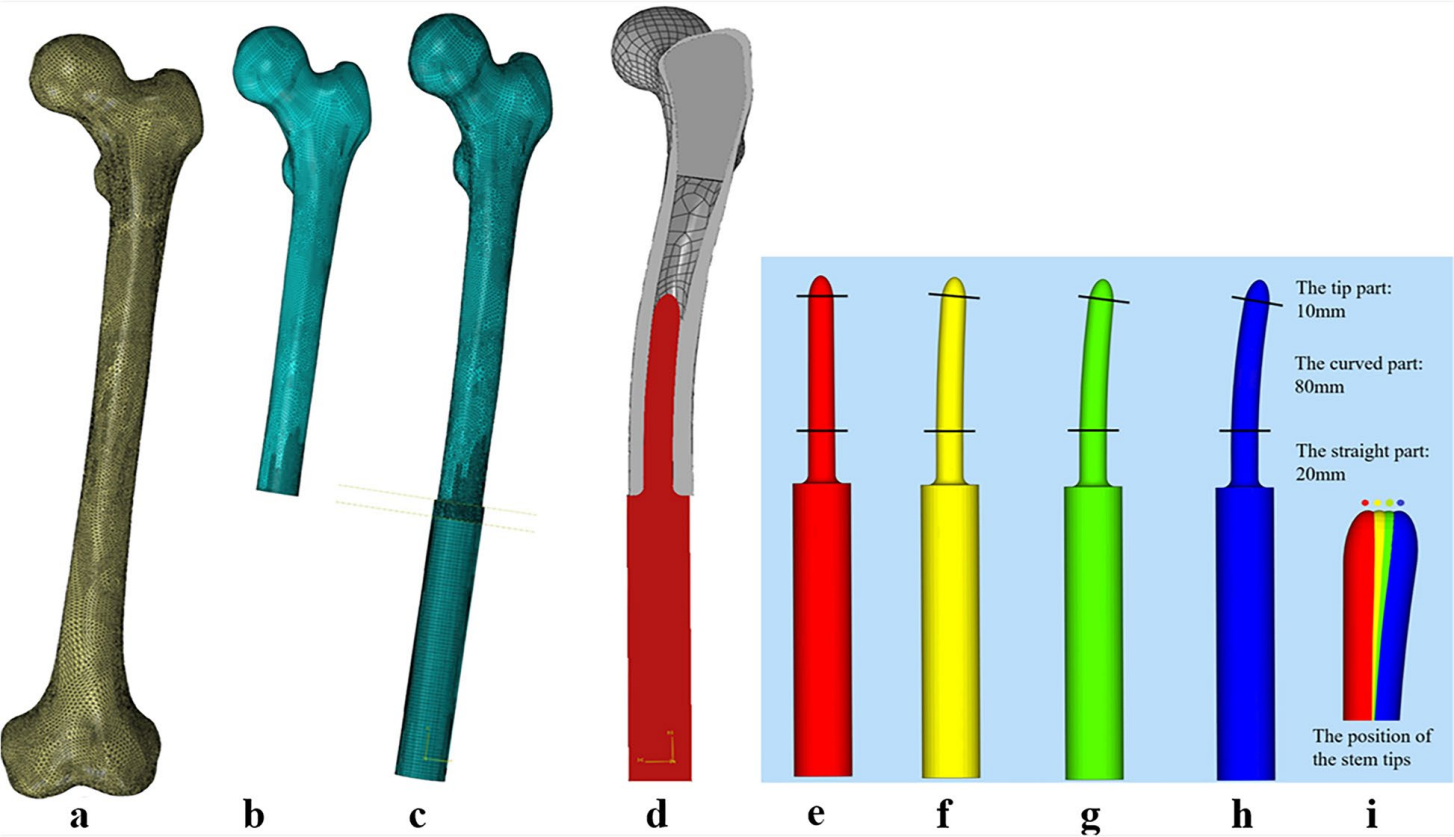
Diagram of the reconstruction models: **a** The normal femur model. **b** The distal tumor resected femora model. **c** The endoprosthetic replacement model. **d** The sectional view of the assembling model. **e** The stright stem. **f, g, h** The curved stems with ROC of 1300 mm, 700 mm, and 475 mm. **i** Different stem ROC corresponds to different location of stem tip where the stem was inserted in the medullary canal of the femur

Based on the measurement results of ROC, the femoral CT–scanning data from one participant with standard parameters, a healthy young male volunteer with the height of 168 cm and the weight of 65 kg, was selected to reconstruct the three–dimensional (3D) model of Chinese standardized normal femur using the software Mimics (Fig. 3a). Then, the normal femur model was subsequently imported to Solidworks 2016 (Dassault Systèmes SolidWorks Corporation, France) to simulate the resection of the distal femur, and 40% of the length of femur (160 mm) was distally resected (Fig. 3b).

#### The design of the femoral stem

The relationship between the stem ROC and the femoral ROC determined the extent of their geometrical fit. Therefore, a straight stem (Fig. 3e) and three representative curved stems with different ROC were reconstructed to better reveal the biomechanical properties of the curved stem. Moreover, since 40% of the length of femur is the most commonly range of osteotomy in distal femoral replacement [3], the present study simulated the femoral osteotomy at this level to increase the representativeness of the study. When the femoral stem is implanted at this level of the medullary cavity, it located at the Segment_2_ with the femoral ROC_234_.

Different stem ROC corresponds to different location of stem tip where the stem was inserted in the medullary canal of the femur (Fig. 3i). Apparently, if a curved stem has a ROC close to ROC_234_, the tip of this curved stem would be placed at the center of the corresponding medullary canal (C_3_) in Segment_2_. On this basis, we firstly designed the curved stem C: its stem tip located at C_3_, and it has a stem ROC of 700 mm (Fig. 3g). Then the curved stem B was designed: its stem tip placed 2 mm posterior to the position where the tip of Stem C was located, and it has a smaller stem ROC of 475 mm (Fig. 3h). Finally, the curved stem D was designed: its stem tip placed 2 mm anterior to the position where the tip of Stem C was located, and it has a larger stem ROC of 1300 mm (Fig. 3f).

Except for the ROC features of each stem, the rest of the prosthetic components are identical. All devices were designed as short stem with a total length of 100 mm, and with appropriate size (11 mm–10 mm) and taper (1/100) to achieve press–fit fixation. The roots of the curved stems were designed as straight cylinder shape to ensure the mechanical stability, thereby preventing prosthetic stem breakage (Fig. 3h).

#### Models assemble

The aforementioned components such as the distal tumor resected femora and the different femoral stems were imported into Solidworks 2016, and were assembled to 4 kinds of endoprosthetic replacement models (Fig. 3d). Model A: The distal tumor resected femora + straight stem A; Model B: The distal tumor resected femora + curved stem B; Model C: The distal tumor resected femora + curved stem C; Model D: The distal tumor resected femora + curved stem D.

#### Material assignment and mesh

The cortical bone behaves like a transversely isotropic material and is insensitive to mechanical loads, so its stiffness was supposed to remain constant during the iteration process. The Young’s modulus (E), shear modulus (G), and Poisson’s ratio (μ) of the cortical bone in longitudinal direction, which was parallel to the z–axis of femur–based coordinate system, were assigned as E_L_ = 16.61 GPa, G_L_ = 4.74 GPa, and μ_L_ = 0.370, respectively. As for the transverse direction, the data were assigned as E_T_ = 9.55 GPa, G_T_ = 3.28 GPa, and μ_T_ = 0.45, respectively [18]. The trabecular bone was assumed to be isotropic with E = 1850 MPa and μ = 0.3[19]. The prosthesis (Ti–6Al–4 V) was set at a Young’s Modulus of 110GPa, with a Poisson’s ratio of 0.3.

The finite element solver Abaqus 6.17 (Dassault Systèmes, Paris, France) was used in geometrical nonlinear simulations for deformation and stress analyses. Tetrahedral elements were used to ensure a good representation of the geometry of the bone. Tetrahedral finite elements with quadratic shape functions (C3D10) along with displacement degrees of freedom were used for the discretization of the femur and the implant (Fig. 3c).

#### Loads and boundary conditions

**Fig. 4:**
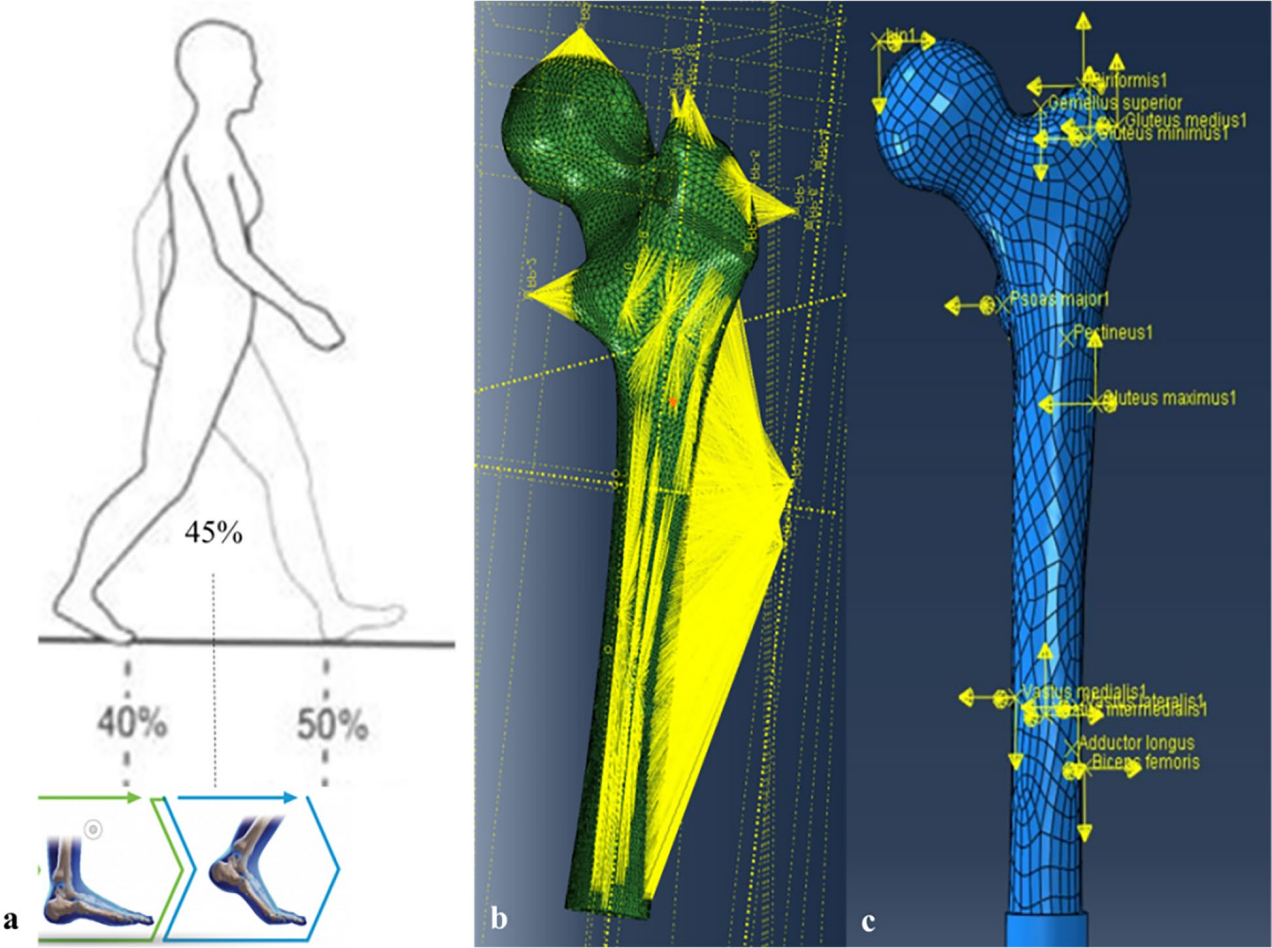
Diagram of the loads and boundary conditions: **a** An instant at forty five percent of the gait cycle. **b, c** The hip joint–femur muscle multiple force was applied to these FE models, with displacement constraints were applied to the condyles or the distal part of the prosthesis

Loads of the magnitudes specified for the 45% position in the gait cycle (push–off during one legged stance and thus a peak load) were applied to all models (Fig. 4a). According to the studies of Viceconti et al. [20, 21], the femoral insertion areas with the center point of major femoral muscles were mapped on the surface of each model. Additionally, the loads were applied as recommended by Duda et al. [22]. Each load was directly applied on the particular mapping center point, which was coupling with the corresponding muscle attachment area and joint contact surface, avoiding artefacts associated with concentrated force application (Fig. 4b). As distal contact forces were not required from our models, displacement constraints were applied to the condyles or the distal part of the prosthesis (Fig. 4c).

In this study, it was assumed that the bone–stem interface was fully integrated, and the relative sliding did not occur under the condition of stress loading. Therefore, the interface between the stem and the medullary canal was set as perfect bonding/sticking friction in Abaqus 6.17 to simulate the mechanical effects of the osseointegration. The contact of the cortical bone–trabecular bone interface was assumed to be tied.

#### Finite element analysis

For the first step, the finite–element model of the normal femur was subjected to a static stress analysis in order to establish the reference state (i.e., the physiologically normal stress and strain environment). After that, an iterative finite–element analysis of the resected distal femurs with implantation of different stems was performed to predict the biomechanical changes that occur due to different setting related to the ROC of stems.

## Results

### The ROC analysis of femur in different segments

Totally, 48 men and 48 women who met the inclusion and exclusion criteria with a mean age of 31.57 years (range, 20–40 years) were included in the anatomical analysis part of this study. The mean height was 170.22 cm (range,160–180 cm), the mean height for females was 164 cm (range, 160–170 cm) and for males was 176.44 cm (range, 168–180 cm). The mean weight was 63.04 kg (range,45–80 kg), the mean weight for females was 56.44 kg (45–65 kg) and for males was 69.65 kg (range, 58–80 kg). The average data of ROC_123_, ROC_234_, ROC_345_, ROC_456_, and ROC_567_ for all volunteers were shown in Table 2.

**Table 2:** The diameter of curvature in sagittal panel of each segment and the full length

Segment	ROC_***_	ROC (means ± SD, mm)
Segment_1_	ROC_123_	724.5 ± 216
Segment_2_	ROC_234_	747.5 ± 203
Segment_3_	ROC_345_	1016.5 ± 440.5
Segment_4_ Segment_5_ Full length	ROC_456_ ROC_567_ ROC_147_	1286.5 ± 383 1128.0 ± 441.5 1127.0 ± 236

The results shown that there were no statistically significant differences between the ROC of Segment_1_ and Segment_2_ (*P* = 0.45), as well as between the ROC of Segment_3_ and Segment_5_ (*P* = 0.957). The magnitudes of the mean values of ROC were in the following order: Segment_1_≈Segment_2_ < Segment_3_≈Segment_5_ < Segment_4_ (*P* < 0.001). The magnitudes of ROC showed a trend of first increasing and then decreasing from the proximal to the distal end of the femoral canal. The variation of the ROC from Segment_2_ to Segment_3_ was relatively large, which indicated that this region has a more significant anteriorly bowed shape than other segments.

### Finite element analysis results

#### The stress distribution on the normal femur

**Fig. 5:**
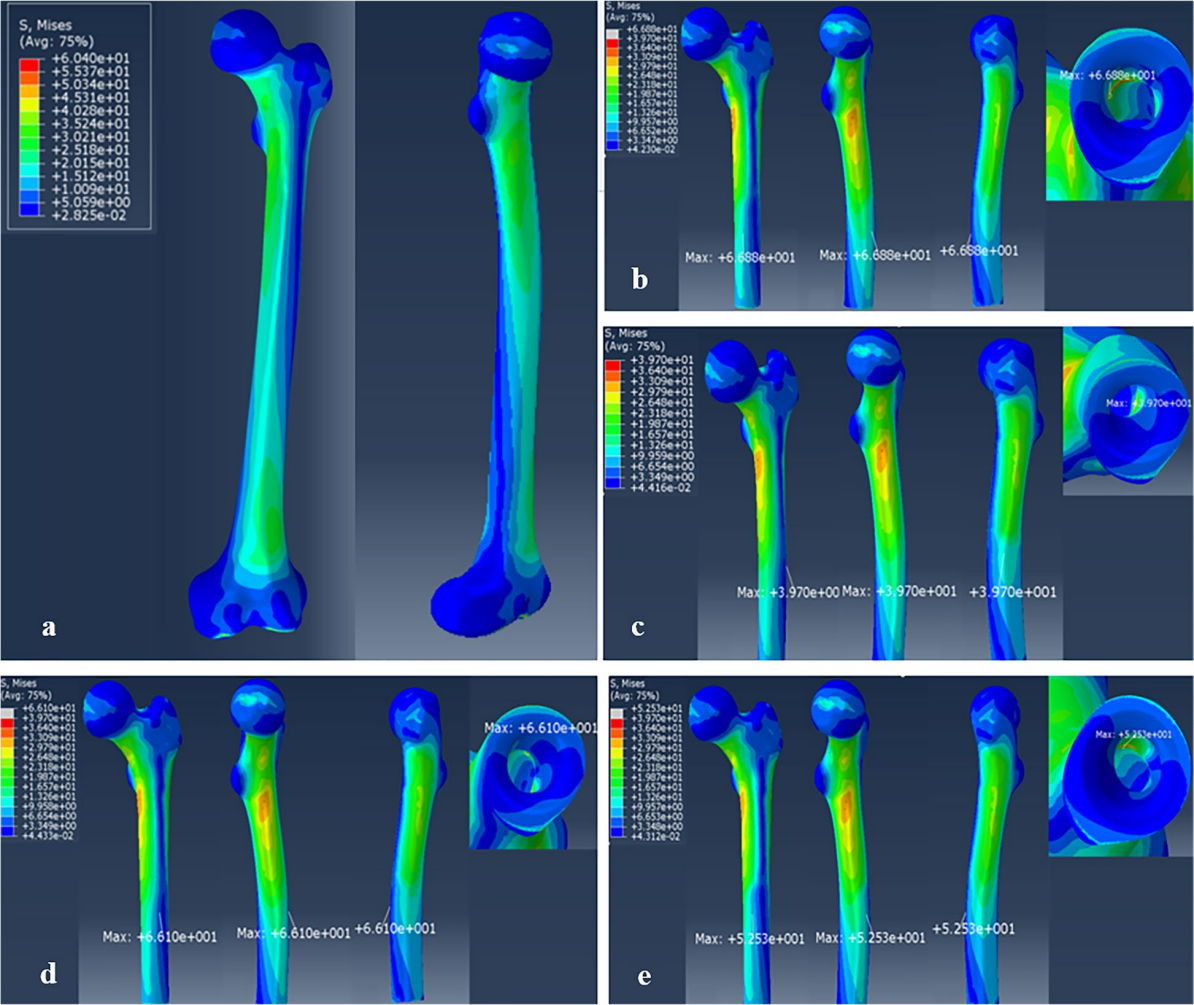
The stress distribution of the normal femur and the femurs after endoprosthetic replacement: **a** The normal femur. **b** The femur reconstructed by the curved stem with ROC of 1300 mm. **c** The femur reconstructed by the curved stem with ROC of 475 mm. **d** The femur reconstructed by the straight stem. **e** The femur reconstructed by the curved stem with ROC of 700 mm

The stress evenly distributed through the whole normal femur, and mainly concentrated on the femoral neck, the lateral and medial sites of the proximal femur, and the anterior site of the distal femur with the stress values up to 35 MPa (Fig. 5a). Generally, the FE results of the normal femur were consistent with those of previous studies [20, 23].

#### The stress distribution on the femurs after endoprosthetic replacement

As an overall perspective, the patterns of stress distribution on the femurs after endoprosthetic replacement (group A, B, C, and D) were similar to the normal femur. The relatively similar level of stress was found on the femoral neck (18–23 MPa), the medial part (13–25 MPa) and the lateral part of each remaining femur (13–22 MPa).

However, the influence of different stems on the mechanical properties is mainly related to the following two aspects: Firstly, varying degrees of stress shielding were observed in the distal remaining femurs after the prosthetic stem implantation (straight stem or curved press–fit stems with different ROC). Compared to the curved stem groups, a more severe stress shielding can be observed in the straight stem group. For the sake of convenience, we defined L as the axial length of the stress shielding area and used this parameter to evaluate the severity of stress shielding. The results shown that L_A_ = 76 mm > L_D_ = 70 mm > L_C_ = 67 mm > L_B_ = 65 mm. Secondly, even though the maximum stress on the femurs of all endoprosthetic replacement models were located at the same corresponding position (stem tip–cortex contact area), the values of the maximum stress were different. We defined S as the maximum von Mises stress of the femur and the results shown that S_D_ = 66.8 Mpa > S_A_ = 66.1 Mpa > S_C_ = 52.5 Mpa > S_B_ = 39.7 Mpa (Fig. 5b–e). The result suggesting that the endoprosthestic replacement using the straight stem and the curved stem with ROC of 1300 mm may be easier to cause damage on the stem tip–anterior cortical area.

#### The stress distribution of different stems

The overall stress distribution of the four different stems were similar. The stress was mainly focused on the anteromedial and posterolateral regions of the stem, and it gradually decreased in a proximal to distal direction along the stem. More specifically, higher stress distributed on the proximal one third of the stem in each model while a lower stress level was recorded on the distal one third of the stem.

**Fig. 6:**
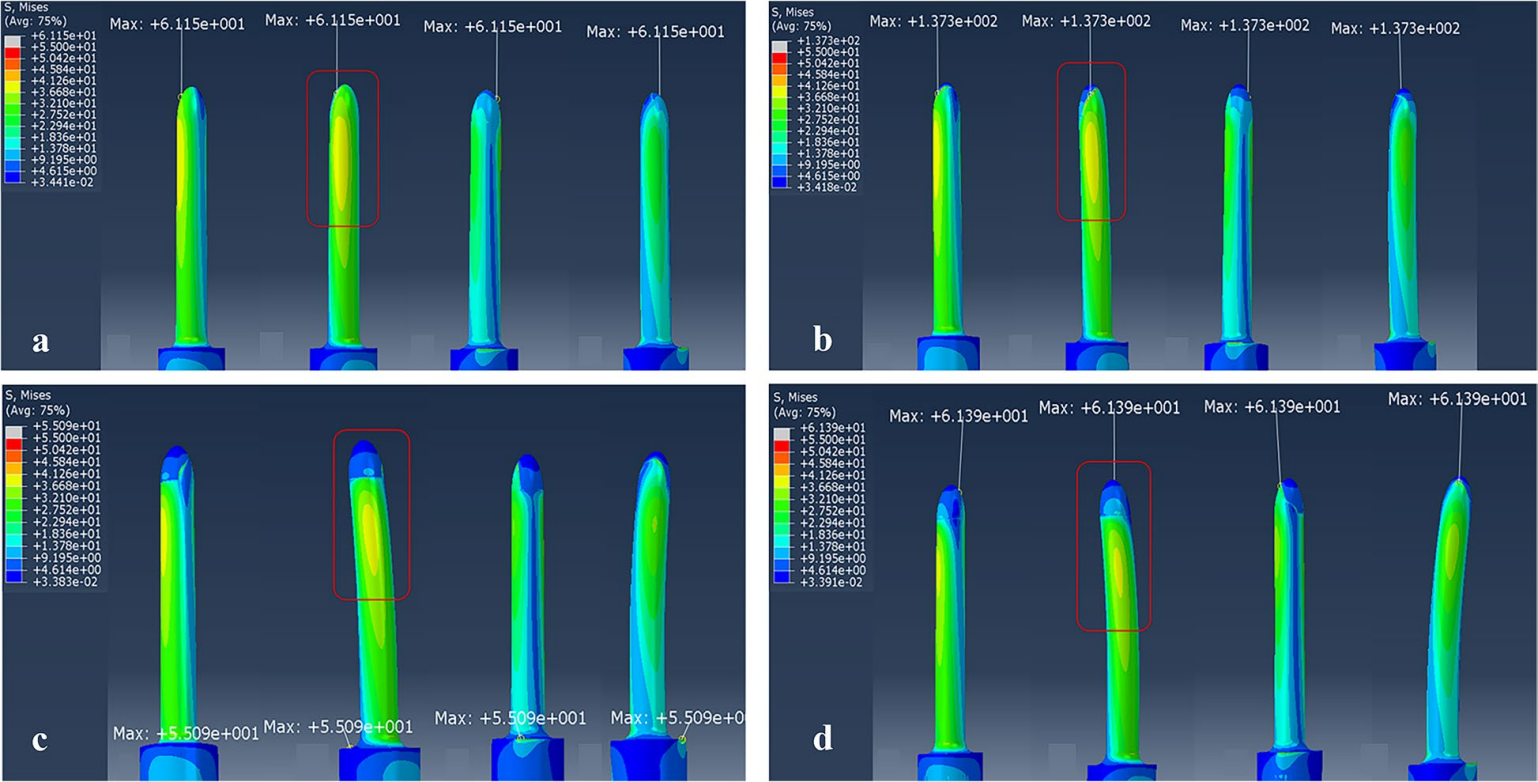
The stress distribution of the femoral stems: **a** The straight stem. **b** The curved stem with ROC of 1300 mm. **c** The curved stem with ROC of 700 mm. **d** The curved stem with ROC of 475 mm

The anterior–medial part of the tip–cortex contact region is a clinically common site for mechanical damage. Except for the curved stem with ROC of 700 mm, the other three kinds of stems’ maximum stress all appeared at this location. However, the peak stress of the curved stem with ROC of 700 mm concentrated on the seat–cortical junction area, where it has a higher machinal strength. We defined M as the maximum von Mises stress of the stems, and the results shown that M_D_ = 137.3 > M_A_ = 61.15 > M_B_ = 61.39 > M_C_ = 55.09. In addition, although the stress mainly concentrated on the proximal one third of the stem for all groups, there was a great variation in the extent of the stress concentration. We assessed the extent of the stress concentration around the proximal one third of the stem based on the area of stress concentration, and the FE results showed that the stress concentration area reached 25% of the high–risk region for group A and D, and 15% for group C, and 10% for group B (Fig. 6a-d).

## Discussion

### The ROC is fundamental in curved prostheses design

Prior studies investigated the anatomical antecurvation of the femoral cavity (i.e., ROC) in different ethnicities [14, 15]. Maratt [24], for example, analyzed the ROC for their sample of 3922 femurs by sagittal reconstruction of the CT scan, and reported that the mean medullary ROC of the full–length femur from Asian American was 1011 mm which is close to ROC_147_ (1127 mm) but far greater than ROC_234_ (747.5 mm) measured in our study. This discrepancy could be explained by the difference in the measurement method. In the study by Maratt, their ROC measurement was performed only to the whole femur, which ignored the morphological differences among different levels of the femoral canal. Abdelaal, et al. [25]. improved the measurement strategy by segmentally measuring the ROC of proximal, middle, and distal thirds of femur. Though analysis of the anatomical data obtained from 132 Japanese femurs, significant differences of ROC were found in different segments, with ROC of proximal femur being the largest (581 mm). However, their study did not take account of the measurement continuity and lacked observations regarding the correlation of anatomical features with the prosthetic design.

In the present study, we made a finer division of measurement segments for the femoral canal and kept an overlapped region between the adjacent segments, ensuring the continuity of data. We found that not only the ROC of Segment_2_ was far below those measured in previous studies, but also the ROC showed a significant change between the Segment_2_ and Segment_3_. Considering that 40% of the length of femur is the most commonly range of osteotomy in distal femoral replacement [3], it is more plausible to distinguish and precisely measure the ROC at this level (i.e., ROC_234_). A curve–designed stem should have a ROC suitable with the femoral canal, which determined the extent of geometrical fit between the curved stem and the residual femoral canal after femoral osteotomy. Therefore, when the femoral curved stem is implanted at Segment_2_, a more suitable morphology of the stem is required. However, it is worth noting that there is wide diversity between the ROC in different parts of a femur and the actual clinical cases may mandate different osteotomy levels (less than or more than 40%). Hence, the curved stem must be tailored according to the patient’s preoperative osteotomy planning and femoral 3D-CT data.

By using a refined segmental measurement strategy, this study compensates for the shortcomings of existing measurement inaccuracy and identified three representative design parameters (475 mm, 700 mm, and 1300 mm) for the curved femoral stem. Finally, a total of three curved stems with different ROC and one straight stem were three–dimensionally reconstructed, and the biomechanical comparison was thus performed. Additionally, for the design of all femoral stems in this study, the short–uncemented stem design was carried out.

### The short stem provides enough mechanical strength and primary stability

According to femoral stem length, they were divided into two types, long stem and short stem, respectively. The implants with short stem were firstly used in the early 1990s, which enable to conserver more bone for subsequent revisions that may be required [26]. Previous studies confirmed that THA with short stem has several advantages compared to that with long stem, such as comparable mechanical strength, less stress shielding risk, convenient implantation, and conservation of bone. Melisik M et al. [27], for example, retrospectively reviewed 17 young patients (younger than 60 years) with femoral neck fractures combined with risk factors who underwent THA with an ultra-short cementless anatomical stem. Satisfied clinical and radiological outcomes were observed, indicating that the use of the short cementless curved stem would be a viable treatment option. Therefore, all prostheses were designed as short stems with a total length of 100 mm in the present study.

From the FEA results, all short stems included the straight type provided a near–normal overall stress distribution in the representative daily activity, the 45% position in the gait cycle, in the remaining femur after endoprosthetic replacement. It suggested that the shorter bone–preserving designs can provide enough mechanical strength and primary stability for the patient’s native bone. The primary stability is crucial for achieving osseointegration, which is of great importance for the durability and longevity of these endoprosthetic stems. An early implant migration usually represents a preamble of aseptic loosening [28]. This situation is analogous to the healing process of bone fracture, where stability plays indispensable roles in healing and for long–term stability. Our results are in agreement with those of Zdero et al. [29] who performed stiffness mechanical tests (axial, lateral, and torsional stiffness) on femoral stems with different stem length to evaluate their mechanical properties, suggesting that the short stem can provide comparable stiffness and strength to the long stem. Moreover, Levadnyi et al. [30] suggested that the short stem has advantage in respect of load transmission and offers a better environment for load transfer to the host bone compared to the long stem. This may partly explain our results that all endoprosthetic replacement models presented a good general stress distribution. However, there are differences in the details in terms of the stress concentration and stress shielding of different FE models, which reflect the mechanical properties of the femoral prosthesis.

### The biomimetic curved stem provided better biomechanical properties than the conventional stem

In the present study, compared to the straight stem, the biomimetic curved stem showed better biomechanical performance both in terms of reducing the extent of the stress shielding of the femur and in terms of minimizing the stress concentration of the implant. These results might be explained by the fact that the curved stem with a suitable ROC provided a better geometric fit between the stem and the medullary cavity of the femur. In contrast, the morphologic mismatch between the straight stem and the femoral canal consequently leads to a stress distribution mismatch, resulting in a more significant stress shielding or a stress concentration effect.

In the first place, regarding the stress shielding effect, even though same trend of the stress distribution was found among four groups, the curved stem group showed less stress shielding in the distal femur. Severe stress shielding can lead to bone loss around the femoral stem, which is considered to be an important contributor to postoperative aseptic loosening due to the insufficient bone supporting the implant. When bone loss occurs around the femoral stem, the bone–implant interface could be exposed to wear particles, thus inducing the implant loosening [31, 32]. Therefore, a lower extent of stress shielding is beneficial to reduce the risk of aseptic loosening. Additionally, another interesting finding is that the FE results of curved stem D were close to those of the straight stem. The curved stem D has a ROC of 1300 mm, and it more closely resembles the straight stem in morphology. This result further indicates that the biomimetic design of the femoral stem has an important role in preventing stress shielding and aseptic loosening.

In the second place, the maximum stress in all groups were appeared in the stem tip–cortex contact area, which has been considered as the most common site of involvement for aseptic loosening. However, the curved stem B and C showed a relatively lower stress concentration level than the straight stem and the curved stem D. Bone loss in this venerable region after stem implantation is commonly attributed to high stress concentration which breaks the balance between bone formation and resorption. Moreover, high stress concentrations in the high–risk regions might be the origin of wear particles which is believed to be the main triggering cause of aseptic loosening. Thus, these results suggested that the load transmission is more physiological in the curved stem with a suitable ROC compared to the straight stem, and the curved stem has a lower risk of prosthetic stem breakage and an improved stress distribution patten.

Taken together, our study confirmed that the endoprosthetic reconstruction using.

knee endoprosthesis with cementless, curved, short stem could provide enough mechanical support and has advantages in reducing the aseptic loosening rate. However, further clinical experiments are required to support these results and to evaluate its clinical application value.

## Limitation and expectation

Our study has limitations. Firstly, this FE analysis was performed only under single–leg support condition, and analysis under other more realistic conditions such as walking, running, and squatting will offer more accurate data in future experiments. Secondly, the nonhomogeneous, inelastic, and nonlinear material of the bone and the implants were overlooked, requiring further research and precise data to test our conclusions. Finally, the curved stem with a ROC of 40% femur length wouldn’t be able to perfectly match the femoral medullary canal whenever the resected percentage is less or more than 40%. Even though 40% femur length represented the most common resection percentage of distal femur in clinic, the conclusion of this study may not apply for those patients whose resected percentage were less or more than 40%. The future work should include stem designing and biomechanical analyzing based on those sites of the femur.

## Conclusion

The uncemented, curved, short stem with suitable ROC can perfectly match the Chinese femoral canal morphology which has better mechanical properties than the conventional femoral stem by decreasing the stress shielding and avoiding the stress concentration. Thus, the endoprosthetic reconstruction using knee endoprosthesis with the uncemented, curved, short stem might have some benefits in decreasing the incidence of aseptic loosening, and it might be an optimized method for treatment of malignant femoral tumours in the Chinese populations in the case that the numerical results are supported by future experimental studies.

## Data Availability

The datasets used and analyzed during the current study are available from the corresponding author on reasonable request.

## Abbreviations

ROC: Radius of curvature
3D–CT: Three–dimensional computed tomography
HJC: Hip–joint center of rotation
sTEA: Surgical transepicondylar axis
FEA: Finite element analysis
